# A Need for Standardization of the Diagnosis and Treatment of Pelvic Inflammatory Disease: Pilot Study in an Outpatient Clinic in Quito, Ecuador

**DOI:** 10.1155/2020/5423080

**Published:** 2020-05-09

**Authors:** Francisco Cueva, Andrés Caicedo, Paula Hidalgo

**Affiliations:** ^1^Instituto de Investigaciones en Biomedicina, Universidad San Francisco de Quito, 17-12-841 Quito, Ecuador; ^2^Colegio de Ciencias de la Salud, Escuela de Medicina, Universidad San Francisco de Quito, 17-12-841 Quito, Ecuador; ^3^Sistemas Médicos, SIME, Universidad San Francisco de Quito, 17-12-841 Quito, Ecuador

## Abstract

**Background:**

Pelvic inflammatory disease (PID) diagnosis is often challenging as well as its treatment. This study sought to characterize the diagnostic and therapeutic trend among physicians at the outpatient level, in Quito, Ecuador, where currently no nationwide screening or specific clinical guideline has been implemented on PID or its main microbiological agents.

**Methods:**

A retrospective analysis of medical records with pelvic inflammatory disease diagnosis in an outpatient clinic was performed. Electronic medical records from 2013 to 2018 with any pelvic inflammatory disease-related diagnoses were retrieved. Information with regard to age, sexually related risk factors, symptoms and physical exam findings, ancillary tests, method of diagnosis, and antibiotic regimens was extracted.

**Results:**

A total of 186 records were included. The most frequent clinical manifestations were vaginal discharge (47%) and pelvic pain (39%). In the physical examination, leucorrhea was the most frequent finding (47%), followed by lower abdominal tenderness (35%) and cervical motion tenderness in 51 patients (27%). A clinical diagnosis was established in 60% of patients, while 37% had a transvaginal sonography-guided diagnosis. Antibiotic treatment was prescribed with standard regimens in 3% of cases, while other regimens were used in 93% of patients. Additionally, an average of 1.9 drugs were prescribed per patient, with a range from 1 to 5, all in different combinations and dosages.

**Conclusions:**

No standardized methods of diagnosis or treatment were identifiable. These findings highlight the need for standardization of the diagnosis and treatment of PID attributed to chlamydial and gonococcal infections.

## 1. Introduction

Pelvic inflammatory disease (PID) is an infectious polymicrobial disorder of the upper genital tract that affects around 4-12% of young women worldwide [1]. This clinical entity can be attributed to a variety of bacteria. *Chlamydia trachomatis* and *Neisseria gonorrhoeae* are identified in one-half to one-third of cases. Other bacteria such as *Mycoplasma genitalium*, *Ureaplasma species*, and fastidious bacterial vaginosis- (BV-) associated bacteria (*Sneathia* (*Leptotrichia*) *sanguinegens*, *Sneathia amnionii*, and *Atopobium vaginae*) and BV-associated bacteria 1 (BVAB1) are emerging as underdiagnosed etiologic agents [2, 3]. Due to its microbiological heterogeneity, one of the main concerns regarding PID is providing an effective treatment. Additionally, the growing multidrug resistance of gonococci and the absence of an antibiotic regimen that proves to be optimal in terms of safety and effectiveness are a challenge for almost every health-care system [1].

Clinical diagnosis can prove challenging given that there is no reliable clinical pattern to differentiate infected from uninfected women, especially since there is a proportion of patients that can be asymptomatic or have subclinical infections [4–6]. Sexually active patients presenting with pelvic pain accompanied by vaginal discharge, intermenstrual or postcoital bleeding, dysuria, lower back pain, and nausea or vomit warrant a high suspicion for PID [4, 7]. On physical exam, uterine, adnexal, or cervical motion tenderness is the minimum criterion to consider PID [7]. Laboratory and imaging studies are generally recommended for clinically severe symptoms or to study other possible differential diagnoses. White blood cell count, erythrocyte sedimentation rate, and C-reactive protein may aid in diagnosis, although alterations are nonspecific [8]. Nucleic acid amplification tests (NAATs) are superior in determining the etiologic agents, particularly in relation to the presence of chlamydia and gonorrhea, with a sensitivity superior to 90% and a specificity of over 99% [9]. Imaging studies like transvaginal ultrasound have been found to have 30% sensitivity and 67% specificity [10]. Ideally, the diagnosis should be determined by a combination of diagnostic laparoscopy and endometrial biopsy, identifying the affected site and the degree of inflammation. However, these procedures are not routinely performed [11].

Due to the highly variable clinical presentation, the Centers for Disease Control (CDC) has recommended a diagnostic criteria system based on the presence of pelvic or lower abdominal pain in women at risk for sexually transmitted infections, with at least one of the physical exam findings listed before [7]. Diagnosis based on this system has an estimated sensitivity of 83% [12]. The specificity of the diagnosis can be increased by findings of at least one of the following: oral temperature of >38.3°C, abnormal cervical mucopurulent discharge or cervical friability, presence of abundant numbers of white blood cells on saline microscopy of vaginal fluid, elevated erythrocyte sedimentation rate, elevated C-reactive protein, or laboratory documentation of cervical infection with *N. gonorrhoeae* or *C. trachomatis* [7].

Owing to the risk of complications of PID and its potential sequelae, such as chronic pelvic pain, infertility, and ectopic pregnancy, clinicians must decide to start treatment promptly [5, 13, 14]. For this reason, diagnostic criteria with high sensitivity and low specificity can be used to detect most of the patients in need for treatment [13]. Initiating antibiotic therapy with a high level of suspicion will not likely affect the clinical course of other potential underlying pathological processes [7]. Currently, the antibiotic regimens recommended are empirical and broad spectrum due to the microbiological profile of this disease. European, CDC, and the WHO guidelines recommend different antibiotic regimens in response to their epidemiological data [7, 15, 16]. This contrast in treatment patterns is important because it highlights the difference in the standard of care related to bacterial resistance patterns at each location.

Even though chlamydia has been shown to be capable of adopting resisting phenotypes in vitro and that there have been reports of resistance to tetracyclines and macrolides, currently it is the antimicrobial resistance of *N. gonorrhoeae* that is of immediate concern [17, 18]. The WHO maintains a surveillance program through the Gonococcal Antimicrobial Surveillance Programme (GASP). In 2016, 17 out of 57 countries reported decreased susceptibility to extended-spectrum cephalosporins and 28 out of 57 reported resistance to azithromycin and 56 out of 59 to ciprofloxacin [19]. This resistance profile indicates that gonococci are becoming harder to treat, leaving a limited spectrum of antibiotics available for use.

It is not uncommon for underresourced countries to lack screening strategies, clinical guidelines, and epidemiological data on this matter. Such is the case of Ecuador, one of the few Latin American countries that do not report to the GASP [20]. The absence of structured local surveillance plans from public or private institutions could underestimate the real burden of these infections for the general population. This study is the first to characterize how physicians are diagnosing PID and the antibiotic regimens most often prescribed in an ambulatory outpatient clinic in Quito, Ecuador. Our ultimate goal through this pilot research is to detect possible errors and pitfalls to ultimately develop clinical recommendations and standardized protocols.

## 2. Materials and Methods

A cross-sectional retrospective study was conducted in an outpatient clinic located in Quito, Ecuador. This center is a private multispecialty clinic of upper-middle class. Electronic medical records from 2013 to 2018 with any pelvic inflammatory disease-related diagnoses from the International Classification Disease-10 (ICD-10) were retrieved, including A54, A56, A74, A73, and N70-N77. Charts that met any of the following exclusion criteria were eliminated from the study:

- Male patients
- Pediatric patients with exception of adolescents older than 12
- Cases where PID was ruled out by physicians after nucleic acid amplification tests (NAATs) for *N. gonorrhoeae* and *C. trachomatis* were negative
- Duplicated and/or incomplete medical records
- Patients who were diagnosed with PID presumptively but did not complete the complementary studies and therefore did not receive antibiotic treatment
- Incorrect and/or inconsistent ICD-10 diagnoses
- Severe PID diagnosis (surgical emergencies, tuboovarian abscess, pregnancy, severe illness, nausea and vomiting or high fever, inability to tolerate an outpatient oral regimen, and the absence of response to oral antimicrobial therapy) [7]

Information regarding age, sexually related risk factors, symptoms and physical exam findings, ancillary tests, method of diagnosis, and antibiotic regimens was extracted from medical records. For the purpose of this study, the method of diagnosis was considered to be clinical when the physician determined a case of PID solely based on history and physical exam, ultrasound-guided when the clinician determined the diagnosis after receiving the imaging study results, or confirmed by laboratory tests or laparoscopy. Validated combinations of antibiotics published in international guidelines are considered “standard regimens.” These data were built into a database on Microsoft Excel; univariate statistical analysis was performed in the same platform, and graphs were constructed with GraphPad Prism V6.

Among patients who received a clinical PID diagnosis, in order to observe the correlation between the symptoms of lower abdominal pain and uterine, adnexal, or cervical motion tenderness, data were coded as “0” if either lower abdominal or tenderness points in the physical examination were absent or “1” if they were present. Spearman correlation test was performed to assess statistical correlation between these two variables; GraphPad Prism V6 was used.

## 3. Results

From the 515 medical records retrieved, 19 of them corresponded to male patients, 181 had incorrect ICD-10 diagnoses, 43 were empty, five had a PID diagnosis ruled out by the attending physician due to negative NAATs, 44 patients had incomplete workup, 34 were duplicated, two women rejected treatment, and one was transferred to an emergency department. After this selection process, a total of 186 records were included in this study, three of which belonged to the same patients that consulted with a new PID episode with at least six months in between.

From the data recorded, the mean age was 31.4 ± 7.6 years, with a range of 16 to 57 years; 83% of women were under 40 years old, and five patients were adolescents, representing 2.68% of the study group (Sup. 1). Sexually related risk factors were not registered in any medical records. With regard to clinical manifestations, the most common was vaginal discharge, which was present in 87 patients (47%) followed by pelvic pain reported in 72 women (39%) and other symptoms (including lower back pain, malaise, nausea, and vomit) in 36 subjects (19%). In the physical examination, leucorrhea was the most frequent finding, seen in 88 patients (47%), followed by lower abdominal tenderness in 65 patients (35%) and cervical motion tenderness in 51 patients (27%). Uterine and adnexal tenderness were present in a lower proportion (17 and 10%, respectively). A complete list of clinical manifestations and physical examination findings is found in Table 1.

**Figure 1:**
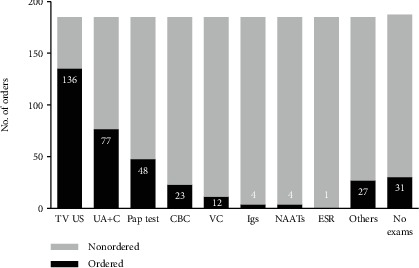
Relative frequency of tests ordered as part of the diagnostic process. In *Y*, we show the number of orders, and in *X*, the type of test. TV US: transvaginal ultrasound; UA+C: urinalysis and/or urine culture; CDC: complete blood count; VC: vaginal culture; Igs: immunoglobulins; NAATs: nucleic acid amplification tests; ESR: erythrocyte sedimentation rate; no exams: no tests ordered.

**Figure 2:**
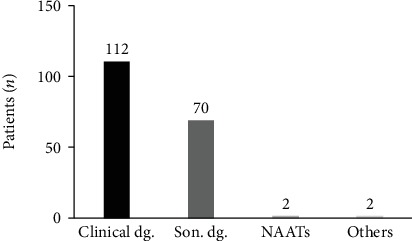
Number of patients diagnosed with pelvic inflammatory disease distributed by diagnostic technique. In *Y*, we present the number of patients, and in *X*, the diagnostic method. Clinical dg.: clinical diagnosis; Son. dg.: sonography-guided diagnosis; NAATs: NAAT-documented infection.

During the diagnostic process, there were 1.9 tests ordered in average for each patient, with a range of zero to seven tests. The test most frequently ordered was transvaginal ultrasound (*n* = 136) (Figure 1). The diagnosis of PID was made based on clinical criteria in 112 patients (60%), while 70 (37%) had an ultrasound-guided diagnosis. Two cases (1%) were documented with NAATs, one patient was diagnosed incidentally through laparoscopic findings, and one was diagnosed with immunoglobulin assays (Figure 2).

From the 112 patients diagnosed with PID clinically, thirteen were excluded from correlation analysis given that their charts did not have a record of physical exam findings. Therefore, out of 99 patients, only 46 patients (46.6%) reported abdominal pain and 66 of them (66.6%) revealed tenderness. There was no statistically significant correlation between the symptoms of lower abdominal pain and uterine, adnexal, or cervical motion tenderness on physical exam (*r* = −0.02; *p* = 0.77).

**Figure 3:**
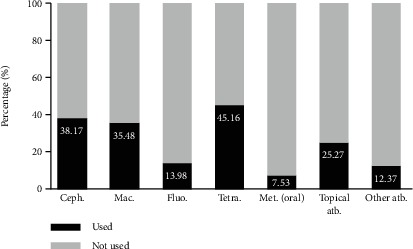
Relative frequency of antibiotics used classified by families. Percentage of treated patients is shown in *Y*; antibiotic family is presented in *X*. Ceph.: cephalosporins; Mac.: macrolides; Fluo.: fluoroquinolones; Tetra.: tetracyclines; Met. (oral): metronidazole (oral); atb.: antibiotics. Topical metronidazole alone or in combination with another topical treatment was included in the “topical antimicrobial” category.

Antibiotic treatment was prescribed with standard regimens in six cases (3%), while other regimens were used in 174 patients (93%). In 6 cases, there was no antibiotic therapy registered. Among the nonstandard antibiotic regimens, many antimicrobial drugs were used (cephalosporins, macrolides, fluoroquinolones, tetracyclines, oral metronidazole, and topical antimicrobials), all of them in different combinations and in various dosages. The number of antibiotics used per patient ranged from 1 to 5 and averaged 1.9 drugs per patient. Sixty-eight patients (36.5%) were treated with only one antibiotic.  Figure 3 summarizes the relative frequency distribution of antibiotics used classified by families.

## 4. Discussion

Identifying patients with PID is challenging for physicians given the heterogeneity of clinical presentation and the fact that prompt and accurate diagnosis can help prevent complications related to this disorder. However, in countries where there is no epidemiological surveillance and clinical guidelines to aid in detecting and treating patients, physicians must adopt foreign recommendations. The present study is the first to characterize how clinicians are diagnosing and treating patients in Quito, Ecuador, a country that currently does not have any public health program implemented concerning PID or sexually transmitted diseases like *C. trachomatis* and *N. gonorrhoeae*, representing a critical need.

This study found young adult women to be most frequently diagnosed with PID, with 83% of patients being under the age of 40. This result is in accordance with other prevalence studies and surveillance programs that report the highest prevalence for PID and chlamydial or gonococcal infections in the age group of 16 to 45 [21, 22].

Although there is no consensus on how to make a clinical diagnosis for PID, there are recommendations such as the CDC criteria mentioned earlier [7]. This study found a large proportion of cases being diagnosed clinically; however, there was no standard to determine what symptoms and clinical signs warranted a PID diagnosis. Without considering the thirteen patients with incomplete clinical records, 46 (46.4%) had the minimum requirement of lower abdominal pain and 66 (66.6%) had uterine, adnexal, or cervical motion tenderness. These two criteria were not significantly correlated (*r* = −0.02; *p* = 0.77). The most common sign and symptom found was vaginal discharge, which can range in sensitivity between 63 and 75% and specificity of 24 to 75% [23]. From these findings, it is evident that clinical diagnosis is being determined regardless of recommended criteria, which makes the diagnosis heterogeneous. This becomes a problem when deciding which patients to treat given that clinicians can potentially be over- or undertreating.

Besides the cases diagnosed clinically, 37% of patients were identified based on ultrasound. However, this imaging study is not considered first-line testing as it is not sensitive or specific enough, especially in uncomplicated PID [10, 23]. Additionally, it is recommended to test for chlamydia and gonorrhea with NAATs, given that the probability of PID increases substantially if these tests are positive. However, because of the etiological heterogeneity of this clinical entity, a negative result does not dismiss the diagnosis [2, 3, 5]. In our study, only 7 patients were tested with NAATs. Five of them were excluded because the attending physician ruled out a PID diagnosis based on negative results. The remaining two cases were considered positive for PID because of reactive NAATs for *N. gonorrhoeae* and/or *C. trachomatis*.

The most striking result was the diversity of antibiotic prescription. Treatment for PID requires the use of broad-spectrum antibiotics to cover chlamydia, gonorrhoeae, and anaerobic bacteria. As fluoroquinolone-resistant strains of *N. gonorrhoeae* emerge, guidelines from organizations such as the CDC or WHO do not recommend the use of fluoroquinolones [7, 16, 24]. Even though Ecuador lacks data on antimicrobial resistance, our study found that almost 14% of patients were treated with fluoroquinolone. Moreover, the WHO recommends initiating dual empirical therapy when there are no data on gonococcal resistance [16]. Our study found that 68 patients (36.5%) were treated with only one antibiotic, even when the vast majority did not have microbiological confirmation to justify the use of only one drug.

The combination of antibiotics found in this study was inconsistent with international guidelines. The 2015 CDC guidelines recommend ceftriaxone 250 mg intramuscularly plus doxycycline 100 mg orally twice a day for 14 days. Ceftriaxone can be substituted for cefoxitin or another third-generation cephalosporin like ceftizoxime or cefotaxime [7]. Another therapeutic alternative is presented by the WHO, suggesting dual therapy with either ceftriaxone 250 mg intramuscular as a single dose or cefixime 400 mg orally once plus azithromycin 1 g orally as a single dose [16]. These standard regimens were only used in 3% of the patients of our study. Finally, 7.53% were treated with metronidazole, but there is no explicit evidence of bacterial vaginosis in the medical records reviewed.

Altogether, these results point at a serious problem for patients with PID. Not only does Ecuador lack antimicrobial resistance information and clinical guidelines, but also clinicians are inconsistently using recommended regimens. Ultimately, patients can develop complications as a result of an incorrect diagnosis or treatment.

This pilot study has several potential limitations. Firstly, data are based on an outpatient clinic that serves mainly upper-middle class patients, which is not representative of the general population. Additionally, a large proportion of charts were excluded, primarily due to inconsistent or incorrect ICD-10 diagnoses. Moreover, electronic medical records often lacked data. For instance, relevant medical history and sexually related risk factors were not reported, which is a key point that would help identify high-risk patients [7]. Interestingly, most patients were married, which would be concerning given that typically sexually transmitted diseases are related to high-risk sexual behavior and that PID cases tend to be more frequent in single, divorced, or separated women [25, 26].

Another limitation is that patients were not followed up after receiving antibiotic therapy; therefore, data on clinical improvement and potential complications is unavailable. Considering the absence of local epidemiological data to guide antibiotic use, follow-up should be mandatory.

To our knowledge, this is the first observational study performed in Ecuador, which serves as an initial insight into the local reality and may lead to further studies extended to the public system and the development of epidemiological strategies.

## 5. Conclusion

In conclusion, we found that physicians are not currently using standardized methods to accurately and systematically detect PID cases nor are they using standardized antibiotic therapies. In the context of these results, the need for standardization of the diagnosis and treatment of pelvic inflammatory disease is evident. Screening, surveillance, and clinical guidelines need to be implemented to make consistent diagnoses and consequently prevent reproductive complications for patients.

## Figures and Tables

**Table 1:** Clinical manifestations and physical examination findings registered in absolute and relative quantities.

Clinical manifestations			Physical examination findings		
	*n*	%		*n*	%
Pelvic pain	72	39	Cervical motion tenderness	51	27
Vaginal discharge	87	47	Adnexal tenderness	18	10
Abdominal pain	28	15	Leucorrhea	88	47
Dyspareunia	25	13	Uterine tenderness	31	17
Amenorrhea	3	2	Cervical ulcer	5	3
Abnormal genital bleeding	14	8	Cervical inflammation	9	5
Urinary symptoms	18	10	Inguinal lymphadenopathy	2	1
Vaginal itching	21	11	Urinary findings	30	16
Recent genital instrumentation	2	1	Lower abdominal tenderness	65	35
Genital foreign body	1	1	Other abdominal tenderness	16	9
Other symptoms	36	19	Other physical findings	28	15

## Data Availability

The data used to support the findings of this study are available from the corresponding author upon request.

## Supplementary Materials

Supplementary Materials Supplementary Figure 1: age distribution in relative frequencies. Ages of the women in the study were organized by frequency. There is a higher proportion of young and middle-age adults in comparison to adolescents. Supplementary Figure 2: marital status absolute frequency distribution. Marital status of patients was recorded and categorized into married, single, divorced, common-law partner, widowed, and not reported (NR). A higher proportion of married patients is evidenced. Supplementary Figure 3: PID diagnosis by clinical specialty. Patients were diagnosed with PID by two main specialities, gynecology and family medicine (Fam. Med), and thirdly by general physicians (Gen. Med).
