# Supplementary material for: A Need for Standardization of the Diagnosis and Treatment of Pelvic Inflammatory Disease: Pilot Study in an Outpatient Clinic in Quito, Ecuador
Source: Infect Dis Obstet Gynecol. 2020 May 9;2020:5423080. doi: 10.1155/2020/5423080 (PMC7232719; doi:10.1155/2020/5423080)
Supplement: Supplementary Materials — Supplementary Figure 1: age distribution in relative frequencies. Ages of the women in the study were organized by frequency. There is a higher proportion of young and middle-age adults in comparison to adolescents. Supplementary Figure 2: marital status absolute frequency distribution. Marital status of patients was recorded and categorized into married, single, divorced, common-law partner, widowed, and not reported (NR). A higher proportion of married patients is evidenced. Supplementary Figure 3: PID diagnosis by clinical specialty. Patients were diagnosed with PID by two main specialities, gynecology and family medicine (Fam. Med), and thirdly by general physicians (Gen. Med). [file 5423080.f1.docx]

**Infectious Diseases in Obstetrics and Gynecology**

***Original article***

A need for standardization of the diagnosis and treatment of pelvic inflammatory disease: pilot study in an outpatient clinic in Quito, Ecuador

Francisco Cueva, MD^1^, Andrés Caicedo, PhD^1,2,3^ Paula Hidalgo, MD^1,2^

^1^Instituto de Investigaciones en Biomedicina, Universidad San Francisco de Quito, 17-12-841 Quito, Ecuador.

^2^Colegio de Ciencias de la Salud, Escuela de Medicina, Universidad San Francisco de Quito, 17-12-841 Quito, Ecuador.

^3^Sistemas Médicos, SIME, Universidad San Francisco de Quito,17-12-841 Quito, Ecuador.

Correspondence: Paula Hidalgo Muñoz; paulahidalgomunoz@gmail.com

**Supplementary Materials**


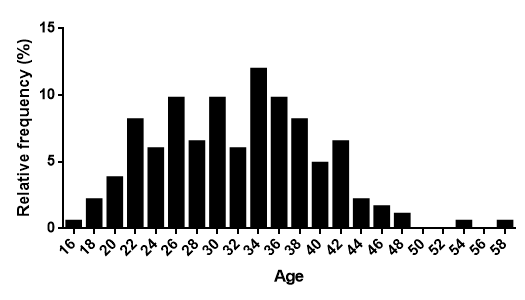


**Supplementary Figure 1. Age distribution in relative frequencies.** Ages from women in the study were organized by frequency. There is a higher proportion of young and middle age adults in comparison to adolescents.

**
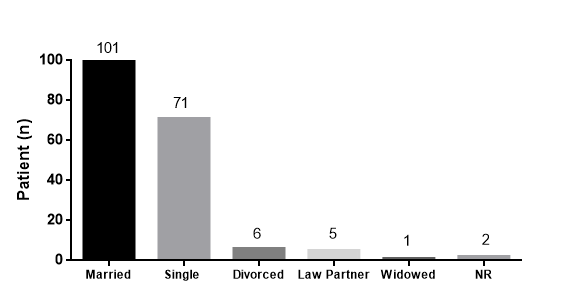
**

**Supplementary Figure 2. Marital status absolute frequencies distribution.** Marital status of patients was recorded and categorized into married, single, divorced, common-law partner, widowed, and not reported (NR). A higher proportion of married patients is evidenced.

**
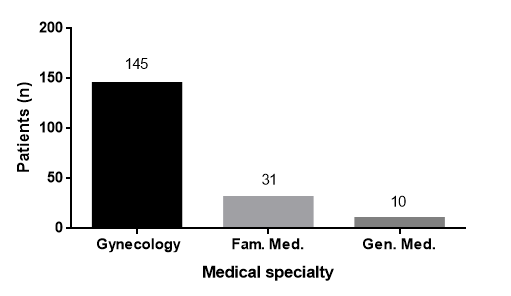
**

**Supplementary Figure 3. PID diagnosis by clinical specialty.** Patients were diagnosed of PID by two main specialites, gynecology and family medicine (Fam. Med), and thirdly by general physicians (Gen. Med).
